# Copper-Based MOF-Derived Core–Shell Materials via N/P/S Ternary Doping for Peroxymonosulfate Activation: Efficient Degradation and Removal of Sulfamethazine

**DOI:** 10.3390/toxics13121023

**Published:** 2025-11-26

**Authors:** Haiyang Zhou, Zhijing Zhang, Shan Zhang, Xiaofeng Wu, Haitao Li, Yong Qiu, Lichao Nengzi

**Affiliations:** 1 College of Resources and Environment, Xichang University, Xichang 615013, China; 2 Academy of Environmental and Economics Sciences, Xichang University, Xichang 615013, China

**Keywords:** sulfamethazine, Cu-MOFs derivative, advanced oxidation processes, reactive oxygen species, peroxymonosulfate

## Abstract

Sulfamethazine (SMT) contamination in aquatic environments and its propensity to induce antibiotic resistance pose critical risks to ecosystems and public health, necessitating effective remediation strategies. Here, we develop a MOF-derived copper core–shell activator, Cu-MOFs400@PSN, by coating a calcined Cu-MOF derivative (Cu-MOFs400) with a nitrogen/phosphorus/sulfur-doped shell and systematically evaluate its peroxymonosulfate (PMS) activation performance toward SMT degradation. Comprehensive characterization confirms successful N/P/S incorporation and the formation of a smooth spherical core–shell architecture that enhances chemical stability; the Cu-MOFs400@PSN/PMS system achieves complete SMT removal within 120 min and maintains 99.33% efficiency after five reuse cycles. Bicarbonate markedly promotes degradation, whereas chloride, nitrate, and phosphate exert negligible interference, indicating strong tolerance to common background ions. Radical-quenching tests identify singlet oxygen (^1^O_2_) and superoxide (•O_2_^−^) as the dominant reactive species, with minor contributions from sulfate and hydroxyl radicals; the system facilitates complete SMT mineralization and reduces resistance-inducing intermediates. These results highlight Cu-MOFs400@PSN as a robust and reusable PMS activator for practical remediation of SMT-contaminated waters and mitigation of antibiotic-resistance risks.

## 1. Introduction

The intensive use and limited biodegradability of sulfonamide antibiotics have led to their frequent occurrence in aquatic environments, fostering the development and spread of antibiotic resistance and posing risks to ecosystems and public health [1,2,3,4]. Sulfamethazine (SMT), a representative sulfonamide, is widely detected and remains challenging for conventional wastewater treatment, motivating advanced oxidation processes (AOPs)—particularly PMS-based systems—for efficient mineralization and compliance with increasingly stringent discharge standards [5,6,7].

Antibiotic resistance (AR) has become a global public health challenge [7], reducing the efficacy of conventional antibiotics and, in some cases, rendering them ineffective, thereby complicating the treatment of infections and increasing mortality [6,7]. As a crucial reservoir and transmission pathway for antibiotics, antibiotic-resistant bacteria (ARB), and antibiotic resistance genes (ARGs), the aquatic environment plays a pivotal role in the occurrence and spread of AR [5,7]. Therefore, developing efficient water treatment technologies to remove antibiotic contaminants and control the transmission of AR is an urgent priority [6,7].

Conventional wastewater treatment processes are often ineffective at removing sulfonamide antibiotics and may fail to meet increasingly stringent discharge standards [8]. Advanced Oxidation Processes (AOPs) are regarded as highly promising water treatment technologies, as they can efficiently and rapidly mineralize organic pollutants into non-toxic small molecules [9,10]. Among them, PMS-based AOPs have attracted extensive attention due to their strong oxidizing capacity, weak pH dependence, and operational simplicity [11,12,13,14,15,16,17,18,19,20]. The activation of PMS is the core of AOPs technology. Efficient activators can effectively enhance the utilization efficiency of PMS and facilitate the progression of the reaction [21,22,23,24].

Metal–organic frameworks (MOFs), as a class of emerging porous materials, have exhibited enormous application potential in the field of catalysis, attributed to their merits such as large specific surface area, tunable pore structure, and abundant metal active sites [25,26,27]. As an important subset of MOFs, copper-based MOFs (Cu-MOFs) have been widely employed in studies on PMS activation for organic pollutant degradation, owing to their low cost and high redox activity [28]. However, conventional Cu-MOFs exhibit poor aqueous stability, which compromises catalytic performance and raises environmental concerns [29,30]. Exploring effective strategies to enhance the stability and catalytic activity of Cu-MOFs remains a key research focus and challenge [31,32,33].

Conventional wastewater treatment often fails to meet discharge requirements for sulfonamide antibiotics, motivating the use of AOPs. Among these, PMS-based systems are attractive due to strong oxidizing capability, weak pH dependence, and operational simplicity. However, Cu-MOF-based PMS activators frequently suffer from inadequate aqueous stability and potential metal leaching, while their long-term reusability and performance under realistic water matrices are often insufficiently demonstrated, limiting practical translation.

Here, we construct a MOF-derived copper core–shell activator, Cu-MOFs400@PSN, by coating a calcined Cu-MOF derivative (Cu-MOFs400) with a ternary N/P/S-doped shell. This architecture enhances chemical stability and preserves Cu^2+^/Cu^+^ redox activity, enabling complete SMT removal with excellent five-cycle durability, rapid and complete removal within 10 min at initial pH 9, and strong tolerance to common anions, where HCO_3_^−^ promotes degradation and Cl^−^/NO_3_^−^/PO_4_^3−^ show negligible effects. Radical-quenching tests identify ^1^O_2_ and •O_2_^−^ as the dominant species, reducing susceptibility to radical scavenging in complex matrices. These advances directly address the prevailing drawbacks of Cu-MOF-based PMS activators—namely aqueous instability, uncertain durability, and insufficient matrix robustness—thereby advancing practical remediation of SMT-contaminated waters.

## 2. Materials and Methods

### 2.1. Instrumentation

Multi-head magnetic heating stirrer (HJ-6), electric blast drying oven (DFZ-6050), electronic balance (FA1204N), ultrasonic cleaner (SL3-300A): Shanghai Sheyan Instrument Co., Ltd. (Shanghai, China). 100 mL hydrothermal reaction kettle: Xi’an Changyi Instrument Equipment Co., Ltd. (Xi’an, China); muffle furnace (SX2-12-10): Dongtai Hongxiang Electric Furnace Mfg. Co., Ltd. (Dongtai, China); high-speed refrigerated centrifuge (TGL-1650): Sichuan Shuke Instrument Co., Ltd. (Chengdu, China); heating reciprocating shaker (HZQ-12OH): Shanghai Yiheng Scientific Instrument Co., Ltd. (Shanghai, China); HPLC (Leaps A10): Beijing Knauer Co., Ltd. (Beijing, China) XRD (X’Pert3 Powder): PANalytical B.V. (Almelo, The Netherlands); XPS (Scientific K-Alpha), SEM (Quattro S): Thermo Fisher Scientific Inc. (Waltham, MA, USA); TGA (STA2500): Shanghai Netzsch Scientific Instrument Trading Co., Ltd. (Shanghai, China); specific surface area/porosity analyzer (ASAP2420): Micromeritics Instrument Corp. (Norcross, GA, USA); TEM (JEM-F200): JEOL Ltd. (Tokyo, Japan); electrochemical workstation (CHI660E): Shanghai Chenhua Instrument Co., Ltd. (Shanghai, China).

### 2.2. Experimental Chemical Reagents

1,3,5-Benzenetricarboxylic acid, copper(II) nitrate trihydrate, absolute ethanol, hexachlorocyclotriphosphazene (Cl_6_N_3_P_3_), 4,4′-dihydroxydiphenyl sulfone, tetrahydrofuran, triethylamine, potassium peroxymonosulfate, sulfamethazine (all AR grade), and methanol (HPLC grade) were from Shanghai Maclin Biochemical Technology Co., Ltd. (Shanghai, China). Phosphoric acid (HPLC grade), L-histidine, sodium bicarbonate, sodium chloride, sodium nitrate, potassium dihydrogen phosphate (all AR grade) were from Chengdu Kelong Chemical Co., Ltd. (Chengdu, China).

### 2.3. Material Preparation Methods

#### 2.3.1. Synthesis of Cu-MOFs400

A colorless transparent solution (Solution A) was obtained by dissolving 1,3,5-benzenetricarboxylic acid (10 g) in absolute ethanol (300 mL). A blue solution (Solution B) was prepared by dissolving copper(II) nitrate trihydrate (20.7 g) in ultrapure water (300 mL). Solution B was combined with Solution A under magnetic stirring for 30 min to yield a homogeneous blue mixture, which was then placed in a 500 mL PTFE-lined stainless-steel autoclave and heated at 110 °C for 24 h. After cooling to room temperature, the supernatant was discarded, and the blue precipitate was soaked in absolute ethanol for 12 h. The solid was recovered by centrifugation (8000 rpm, 10 min, 25 °C), washed three times with ethanol and three times with ultrapure water, and dried at 60 °C for 6 h to obtain a light-blue solid (Cu-MOFs). The obtained Cu-MOFs were subsequently calcined in a muffle furnace at 400 °C for 3 h to yield a black solid (Cu-MOFs400).

#### 2.3.2. Synthesis of Cu-MOFs400@PSN

The synthesis method of Cu-MOFs400@PSN is as follows [34]. In a 250 mL beaker, hexachlorocyclotriphosphazene (0.3 g) and 4,4′-dihydroxydiphenyl sulfone (0.675 g) were dissolved in a mixed solvent of ethanol/tetrahydrofuran (150 mL, 1:1, *w*/*w*) by sonication for 5 min. Cu-MOFs400 (1.0 g) was introduced and sonicated for 10 min to ensure uniform dispersion. Triethylamine (30 mL) was subsequently added in three aliquots (10 mL each), the vessel was sealed with polyethylene film, and the mixture was stirred magnetically for 8 h. The resulting solid was recovered by centrifugation (8000 rpm, 10 min, 25 °C), washed four times with ethanol and four times with ultrapure water, and dried at 55 °C for 12 h to yield a gray-white core–shell composite (Cu-MOFs400@PSN). Drying above 60 °C may induce yellow by-product formation.

### 2.4. Degradation Experiment

Add 100 mL of 10 mg L^−1^ SMT solution and PMS to a 250 mL conical flask, then add Cu-MOFs400@PSN (10–60 mg as specified). Conduct reactions in a thermostatic shaker (25 °C, 180 rpm). Each condition was tested in triplicate.

## 3. Results and Discussion

### 3.1. Material Characterization and Analysis

**Figure 1:**
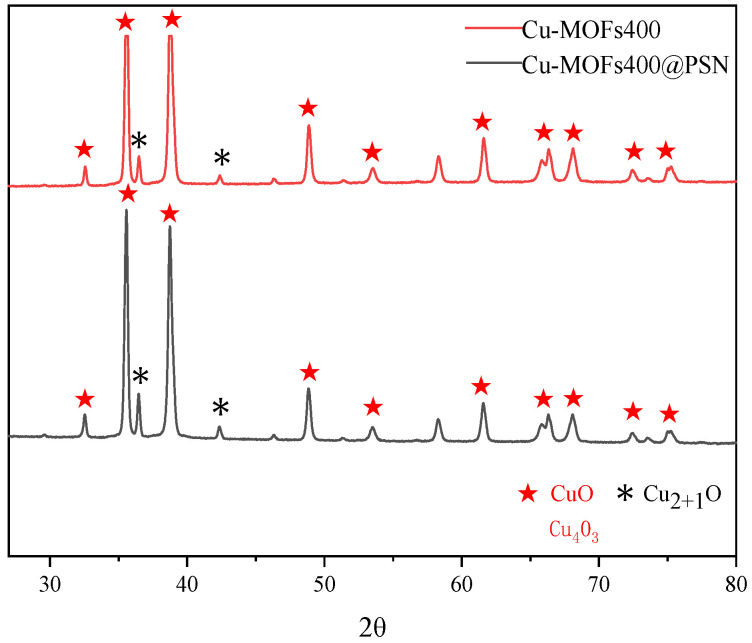
XRD patterns of activated materials.

The Cu-MOFs400@PSN material exhibits excellent stability, which may be associated with the structural modification of Cu-MOFs400 induced by nitrogen (N), phosphorus (P), and sulfur (S) atoms. XRD analysis was employed to characterize the phase and crystalline structures of Cu-MOFs400 and Cu-MOFs400@PSN. The corresponding results are presented in Figure 1.

The XRD patterns of Cu-MOFs400 and Cu-MOFs400@PSN are essentially identical, with diffraction peaks assigned to CuO (PDF#48-1548), Cu_2-x_O (PDF#05-0667), and Cu_4_O_3_ (PDF#49-1830), indicating that Cu mainly exists in these phases [35,36]. No distinct peaks for N, P, or S species were observed, suggesting that dopants are highly dispersed and induce structural modulation without forming separate crystalline phases.

**Figure 2:**
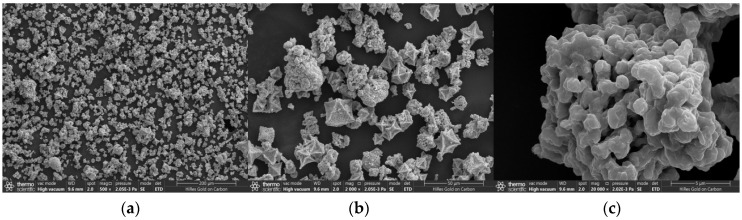

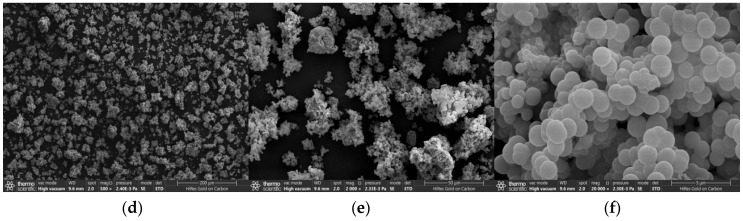
SEM images of activated materials; (**a**–**c**) Cu-MOFs400, (**d**–**f**) Cu-MOFs400@PSN.

To investigate whether the doping of N, P, and S atoms into Cu-MOFs leads to the formation of the PSN shell layer, SEM was employed to characterize the morphological structures of Cu-MOFs400 and Cu-MOFs400@PSN. The corresponding results are presented in Figure 2.

Calcined Cu-MOFs400 exhibits a porous octahedral morphology, whereas Cu-MOFs400@PSN displays a smooth spherical morphology with dense aggregation, indicating encapsulation by the PSN shell. This phenomenon indicates that the Cu-MOFs400 is encapsulated by the PSN shell layer, and N, P, and S are doped onto the surface of Cu-MOFs400—leading to the structural transformation of Cu-MOFs400 from octahedron to sphere.

**Figure 3:**
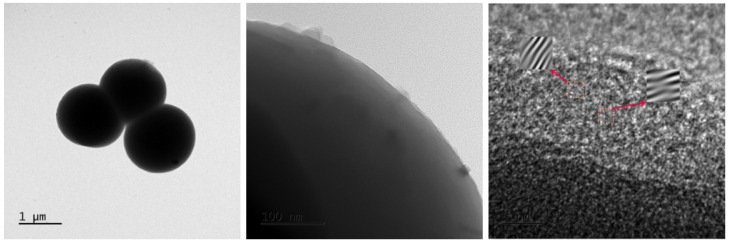
TEM image of Cu-MOFs400@PSN. The red arrows indicate the schematic diagram of local magnification positions.

To further investigate the effect of the PSN shell layer on the structure of Cu-MOFs400, TEM was employed to analyze the unit cell structure of Cu-MOFs400@PSN, and the results are presented in Figure 3.

TEM images of Cu-MOFs400@PSN show sparse and locally curved lattice fringes with uneven spacing, consistent with structural modification induced by the PSN shell.

**Figure 4:**
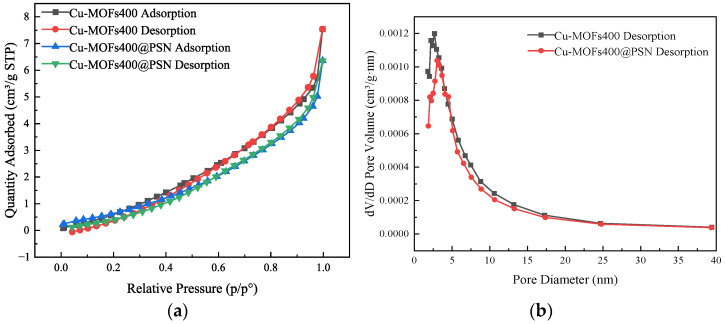
BET analyses of Cu-MOFs400 and Cu-MOFs400@PSN; (**a**) Adsorption–Desorption Isotherm, (**b**) Pore Size Distribution Plot.

The encapsulation of Cu-MOFs by the PSN shell layer can alter the specific surface area and surface pores of Cu-MOFs. To analyze these properties (specific surface area and pores) of Cu-MOFs400 and Cu-MOFs400@PSN, a fully automatic specific surface area and porosity analyzer (BET) was employed, and the corresponding results are presented in Figure 4.

**Figure 5:**
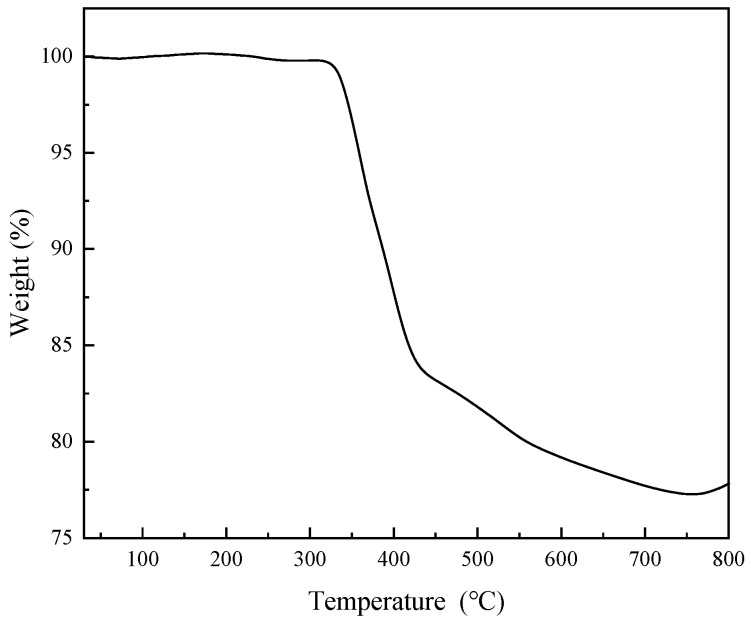
TG profile of Cu-MOFs400@PSN.

Both materials exhibit H3-type isotherms with pore sizes of 1–40 nm, indicating mesoporosity [37]. Specifically, Cu-MOFs400 has a specific surface area of 5.2344 m^2^/g and an average pore size of 5.4682 nm, while Cu-MOFs400@PSN shows a specific surface area of 3.1161 m^2^/g and an average pore size of 5.7228 nm. These results suggest structural reorganization during core–shell formation, merging some micropores into larger mesopores. Consequently, Cu-MOFs400@PSN exhibits a reduced specific surface area and an increased average pore size [38,39]. To investigate the stability of the PSN shell layer and the [parent] material, TG was employed to evaluate the thermal stability of the core–shell material, with the results presented in Figure 5.

TGA reveals three stages: 30–320 °C (stable, negligible mass loss), 320–420 °C (~15.23% loss, attributed to PSN shell detachment), and 420–800 °C (~6.95% loss, decomposition of Cu-MOFs400), indicating good stability below 300 °C.

**Figure 6:**
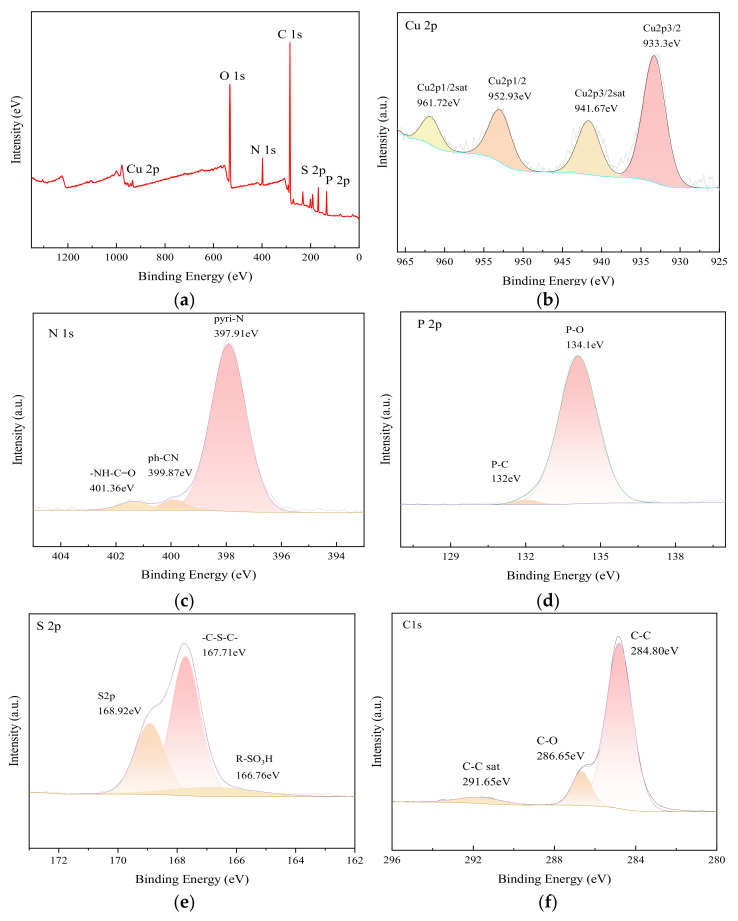
XPS spectra of Cu-MOFs400@PSN; (**a**) Survey spectrum, (**b**) Cu 2p high-resolution spectrum, (**c**) N 1s high-resolution spectrum, (**d**) P 2p high-resolution spectrum, (**e**) S 2p high-resolution spectrum, (**f**) C 1s high-resolution spectrum.

After Cu-MOFs400 is encapsulated by the PSN shell layer, the chemical species in the material undergo changes. To analyze the chemical elements and their chemical valence states in Cu-MOFs400@PSN, X-ray photoelectron spectroscopy (XPS) was employed, with the results presented in Figure 6.

XPS shows Cu 2p_3/2_ and Cu 2p_1/2_ features consistent with Cu^2+^ and Cu^+^, respectively, along with corresponding satellite peaks; N 1s peaks at 397.91, 399.87, and 401.36 eV (pyridinic N, nitrile N, and amide N), P 2p at 132.0 and 134.1 eV (P–C and P–O), and S 2p at 166.76, 167.71, and 168.92 eV (R–SO_3_H, C–S–C, and oxidized S), confirming N/P/S incorporation.

**Figure 7:**
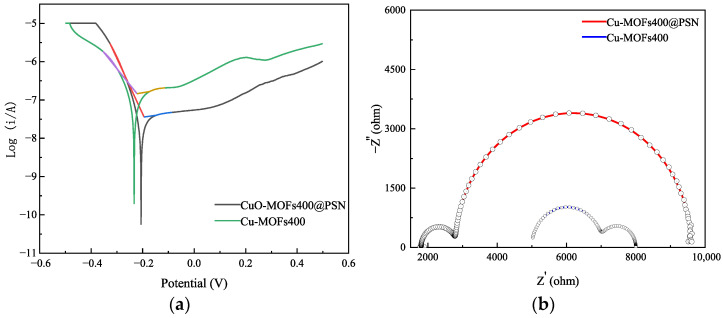
Electrochemical analysis; (**a**) Tafel plots, (**b**) Electrochemical impedance spectroscopy plots.

Structural and morphological characterizations were conducted to reveal the physical and chemical properties of Cu-MOFs400 and Cu-MOFs400@PSN. Meanwhile, the electrochemical performances of the two materials were evaluated via LSV, Tafel plots, and EIS. The corresponding results are presented in Figure 7.

The open-circuit potentials for Cu-MOFs400 and Cu-MOFs400@PSN are approximately 0.22 V and 0.19 V, respectively; the more positive OCP indicates better corrosion resistance for Cu-MOFs400@PSN. The lower corrosion current density also supports enhanced anti-corrosion performance of the core–shell material.

For the charge transfer behavior: Cu-MOFs400 shows a relatively low real impedance (Z’). A low Z’ value implies a small charge transfer resistance (Rct) and high charge transfer efficiency, rendering Cu-MOFs400 suitable for applications requiring rapid charge transfer. EIS shows a larger real impedance (Z’) and charge-transfer resistance (Rct) for Cu-MOFs400@PSN, attributable to the shell-induced structural modulation, making it suitable for controlled, slower charge transfer.

### 3.2. Effect of Reaction Systems on SMT Degradation Efficiency

**Figure 8:**
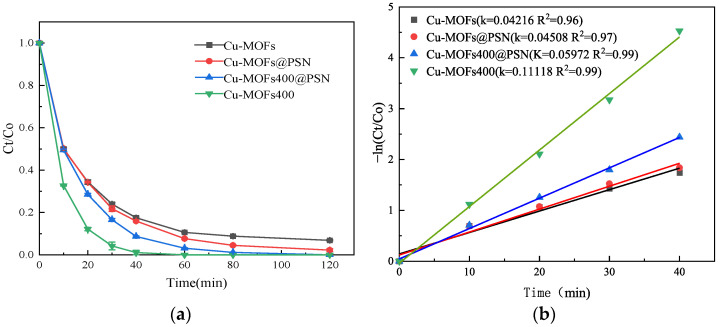
Effect of Reaction Systems on SMT Degradation Efficiency; (**a**) Influence of Degradation Efficiency/Effect on Degradation Efficiency, (**b**) Kinetics Analysis.

Cu-MOFs, Cu-MOFs@PSN, Cu-MOFs400, and Cu-MOFs400@PSN exhibited distinct PMS activation efficiencies, leading to different SMT degradation performances. Unless otherwise stated, the conditions were: SMT 10 mg L^−1^, volume 100 mL, initial pH 5.7, PMS 70 mg, 25 °C, 180 rpm. Equal volumes of the reaction solution and PMS were dispensed into four 250 mL conical flasks. Concurrently, 40 mg of Cu-MOFs, Cu-MOFs@PSN, Cu-MOFs400, and Cu-MOFs400@PSN were individually added to each flask. Samples were collected at reaction time intervals of 0, 10, 20, 30, 40, 60, 80, and 120 min to quantify the residual SMT concentration. Each activator-specific experiment was conducted in triplicate (3 parallel replicates). The influence of reaction systems constructed with different activating materials on SMT degradation efficiency was systematically investigated, and the corresponding results are presented in Figure 8.

As shown in Figure 8, all systems (Cu-MOFs/PMS, Cu-MOFs@PSN/PMS, Cu-MOFs400/PMS, and Cu-MOFs400@PSN/PMS) exhibited high SMT degradation efficiencies. The degradation rates varied significantly within the first 40 min, in the order of Cu-MOFs400/PMS > Cu-MOFs400@PSN/PMS > Cu-MOFs@PSN/PMS > Cu-MOFs/PMS. After 40 min, the degradation rate slowed down. When the reaction time reached 120 min, the SMT removal efficiencies of the reaction systems constructed with Cu-MOFs/PMS and Cu-MOFs@PSN/PMS both exceeded 85%. Among them, the Cu-MOFs@PSN core–shell material demonstrated better PMS activation performance. This is mainly because the water stability of Cu-MOFs is insufficient, and the introduction of the core–shell structure can improve water stability, thereby ensuring PMS activation performance [40,41]. The SMT removal efficiencies of the reaction systems constructed with Cu-MOFs400/PMS and Cu-MOFs400@PSN/PMS both reached 100%. Within 60 min, Cu-MOFs400/PMS showed faster SMT removal than Cu-MOFs400@PSN/PMS, likely because the active Cu-MOFs400 content is lower in the core–shell composite [42,43]. After 80 min, the removal efficiencies converged, indicating that the core–shell structure enhances the activation efficiency of Cu-MOFs400 over longer reaction times. The Cu-MOFs400@PSN/PMS system exhibited superior SMT degradation performance, achieving 100% removal. These results suggest that with the appropriate increase in the amount of shell material, Cu-MOFs and Cu-MOFs@PSN/PMS exhibit more efficient SMT degradation performance.

**Figure 9:**
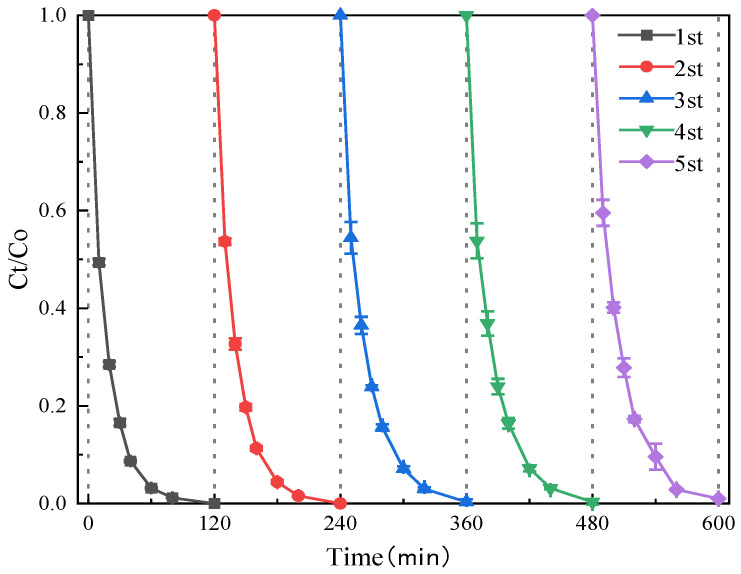
Cyclic Reuse Experiment Figure of Cu-MOFs400@PSN.

The reaction system constructed with Cu-MOFs400@PSN/PMS exhibited superior efficiency in the degradation of SMT. To investigate the degradation stability of Cu-MOFs400@PSN toward SMT, cyclic reuse experiments were conducted on Cu-MOFs400@PSN. The corresponding results are presented in Figure 9.

Cu-MOFs400@PSN exhibited excellent stability over five reuse cycles. When the reaction time reached 120 min, the initial degradation efficiency of SMT by the Cu-MOFs400@PSN/PMS system was 100%. After the fifth cycle, the degradation efficiency still remained at 99.33%, with a decrease of less than 1% [44,45]. This indicates that the active sites of the material (e.g., Cu^2+^ activation centers) did not undergo significant deactivation during the cyclic process. The core–shell material demonstrated high stability, enabling repeated reuse for multiple times and thus reducing the operational cost [46].

### 3.3. Influence of Reaction Conditions on the Degradation and Removal of SMT

**Figure 10:**
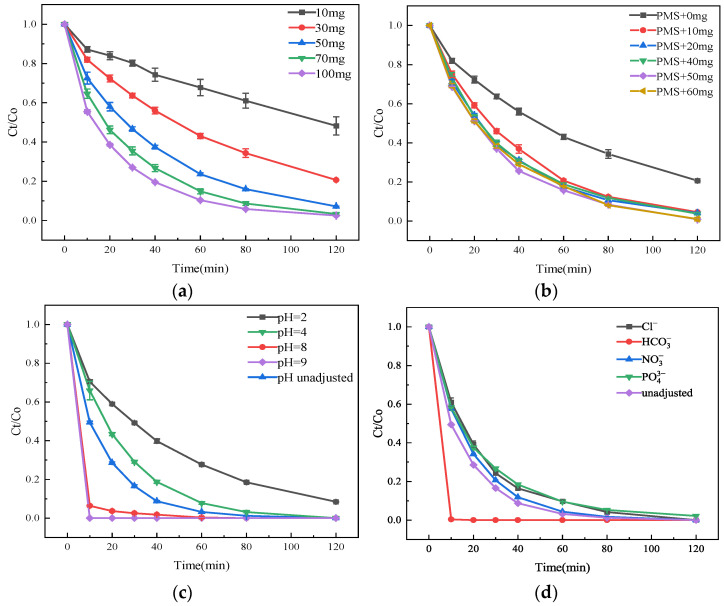
Influence of Reaction Conditions on the Degradation and Removal of SMT; (**a**) The dosage of PMS, (**b**) the dosage of Cu-MOFs400@PSN, (**c**) the pH value of the solution, (**d**) common ions in natural water bodies.

In the Cu-MOFs400@PSN/PMS system, factors including the dosage of PMS, the dosage of Cu-MOFs400@PSN, the pH value of the solution, and the complex matrix of natural water bodies may affect the free radical effect, thereby influencing the degradation efficiency of SMT by the Cu-MOFs400@PSN/PMS system, as illustrated in Figure 10.

As shown in Figure 10a, with PMS = 10 mg, SMT removal was 51.80% at 120 min; increasing PMS from 10 to 70 mg improved removal from 51.80% to 96.72%, while a further increase to 100 mg yielded only a slight increase to 97.52%, indicating excess PMS. As shown in Figure 10b, without catalyst, SMT removal was 79.4% at 120 min; adding 10 mg of Cu-MOFs400@PSN increased removal to 95.25%, and further increasing the dosage to 50 mg improved removal to 99.12%, with a slight decline at 60 mg (99.01%) due to potential ROS scavenging or agglomeration. As shown in Figure 10c, at pH 2 the system achieved 91.60% removal at 120 min, whereas raising pH to 8 markedly accelerated degradation, and pH 9 achieved complete removal within 10 min, indicating strong pH dependence and enhanced PMS activation/electron transfer under alkaline conditions. Balancing efficiency and kinetics, pH 9 was optimal.

As shown in Figure 10d, HCO_3_^−^ led to complete removal within 10 min, suggesting a promoting role, whereas Cl^−^, NO_3_^−^, and PO_4_^3−^ caused negligible suppression (>97% removal at 120 min). This outcome confirms that HCO_3_^−^ ions not only participate in the SMT degradation pathway driven by Cu-MOFs400@PSN-activated PMS but also exert a marked promotional effect on the reaction. The accelerating role of HCO_3_^−^ is presumably attributed to its function as a radical mediator: it facilitates the conversion of primary reactive oxygen species (ROS, e.g., SO_4_^−^) generated from PMS activation into secondary radicals (e.g., HCO_3_^−^ and CO_3_^−^), which exhibit higher reactivity toward SMT than SO_4_^−^ under relevant reaction conditions, thereby boosting overall degradation kinetics. In contrast, when Cl^−^, NO_3_^−^, or PO_4_^3−^ ions were introduced into the Cu-MOFs400@PSN/PMS system, the SMT degradation rate remained above 97% after 120 min of reaction, with no significant deviation in the system’s degradation efficiency. This observation indicates that Cl^−^, NO_3_^−^, and PO_4_^3−^ ions exert negligible interference on the Cu-MOFs400@PSN/PMS-mediated SMT degradation process. Such robust tolerance to common background ions likely stems from the strong PMS activation capacity of Cu-MOFs400@PSN—this material ensures the sustained generation of sufficient ROS even in the presence of these ions, while the ions themselves show low reactivity toward the generated radicals or minimal affinity for the Cu-based active sites on the MOF surface (which would otherwise hinder PMS activation). Collectively, these results underscore the excellent anti-interference performance of the Cu-MOFs400@PSN/PMS system against typical coexisting ions in natural water bodies. Consequently, Cu-MOFs400@PSN demonstrates substantial potential for practical application in the remediation of SMT-contaminated complex aqueous matrices—addressing a key challenge in the translation of advanced oxidation processes (AOPs) from lab-scale to real-world water treatment.

**Figure 11:**
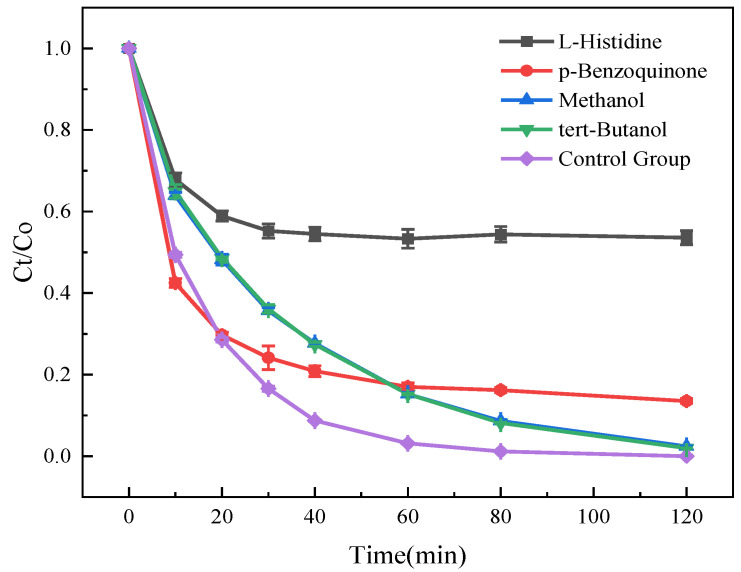
Free Radical Quenching Experiment.

In PMS-based AOPs, key ROS include •SO_4_^−^, •OH, ^1^O_2_, and •O_2_^−^. These reactive species are known to exert significant influences on the degradation efficiency of SMT by the Cu-MOFs400@PSN/PMS system, as illustrated in Figure 11.

As shown in Figure 11, quenching ^1^O_2_ (L-histidine) and •O_2_^−^ (p-benzoquinone) reduced SMT removal to 46.39% and 86.45% at 120 min, indicating their dominant roles [43,47]. In contrast, quenching •SO_4_^−^ (methanol) and •OH (t-BuOH) had minor effects (97.63% and 98.00% at 120 min), suggesting limited contributions from these radicals under the present conditions [48,49].

## 4. Conclusions

This study systematically evaluated the performance and mechanism of Cu-MOFs400@PSN for PMS activation and SMT degradation. The core–shell structured Cu-MOFs400@PSN not only enhances the PMS activation efficiency of Cu-MOFs400 but also actively participates in the PMS-driven SMT degradation process. Notably, the material exhibited excellent stability, maintaining >99% removal after five reuse cycles (120 min). This superior reusability highlights its potential for long-term practical applications in water treatment. Optimal conditions included PMS = 70 mg, Cu-MOFs400@PSN = 40–50 mg, and initial pH = 9, achieving complete removal with fast kinetics. This optimal parameter combination provides a reliable technical basis for the scale-up application of the system in real-world water remediation scenarios. Regarding the influence of coexisting ions in natural water bodies, HCO_3_^−^ significantly accelerated degradation, while Cl^−^, NO_3_^−^, and PO_4_^3−^ had negligible impacts, indicating strong anti-interference capability in complex matrices. Quenching experiments revealed that ^1^O_2_ and •O_2_^−^ dominated the degradation pathway, whereas •SO_4_^−^ and •OH played minor roles under the studied conditions. This finding clarifies the core reaction pathway of the system and provides theoretical support for its performance optimization. Collectively, the Cu-MOFs400@PSN/PMS system integrates high degradation efficiency, excellent structural stability, and strong anti-interference ability, rendering it a promising candidate for the remediation of SMT-polluted water environments. To accelerate translation toward engineering applications, future work should undertake scale-up and validation studies focusing on integration with continuous-flow reactors; controllable synthesis to precisely regulate shell thickness and dopant ratios; and deep removal of trace antibiotic resistance markers in real wastewater.

A limitation of this work is the absence of a dedicated leaching analysis; however, the five-cycle reusability (>99% removal at 120 min) and multi-scale evidence of structural stability support the robustness of Cu-MOFs400@PSN under the tested conditions. Future studies will quantify metal leaching and assess potential risks under extended operation and complex water matrices.

## Data Availability

The data presented in this study are available on request from the corresponding author.

## Abbreviations

The following abbreviations are used in this manuscript:

SMT	Sulfamethazine
PMS	peroxymonosulfate
Cu-MOFs400@PSN	core–shell structured activator
N/P/S	Nitrogen–phosphorus–sulfur
AR	Antibiotic resistance
ARB	antibiotic-resistant bacteria
ARGs	antibiotic resistance genes
AOPs	Advanced Oxidation Processes
MOFs	Metal–organic frameworks
